# Case Report: An Adult Case of Poretti-Boltshauser Syndrome Diagnosed by Medical Checkup

**DOI:** 10.1007/s12311-024-01673-2

**Published:** 2024-02-29

**Authors:** Kensuke Ikeda, Ayane Tamagake, Takafumi Kubota, Rumiko Izumi, Tatsuo Yamaguchi, Kumiko Yanagi, Tatsuro Misu, Yoko Aoki, Tadashi Kaname, Masashi Aoki

**Affiliations:** 1 Department of Neurology, Tohoku University Hospital, Sendai, Japan; 2 Department of Neurology, Tohoku University Graduate School of Medicine, 1-1 Seiryo-Machi, Aoba-Ku, Sendai, 980-8574 Japan; 3 Department of Medical Genetics, Tohoku University Graduate School of Medicine, Sendai, Japan; 4 Department of Radiology Diagnostic Imaging Center, Sendai Seiryo Clinic, Sendai, Japan; 5 Department of Genome Medicine, National Center for Child Health and Development, Tokyo, Japan

**Keywords:** LAMA1, Poretti–boltshauser syndrome, Cerebellar dysplasia, Cerebellar cyst, Whole-exome sequencing

## Abstract

This report describes an adult case of Poretti–Boltshauser syndrome (PTBHS) and with novel variants of *LAMA1*. A 65-year-old Japanese woman with cerebellar malformation identified during a medical checkup was referred to our hospital. Subsequently, neurological examination, brain imaging, and genetic investigation via whole-exome sequencing were performed. The patient presented with mild cerebellar ataxia and intellectual disability. Magnetic resonance imaging revealed cerebellar dysplasia and cysts and an absence of molar tooth sign. Genetic analysis revealed a novel homozygous variant of c.1711_1712del in *LAMA1* (NM_005559.4). Most cases with PTBHS are reported in pediatric patients; however, our patient expressed a mild phenotype and was undiagnosed until her 60 s. These findings suggest that PTBHS should be considered in not only pediatric cerebellar dysplasia but also adult cerebellar ataxia with mild presentation.

## Introduction

Cerebellar hypoplasia is a common finding associated with chromosomal aberrations, metabolic disorders, genetic syndromes, and cerebral malformations. Poretti–Boltshauser syndrome (PTBHS) is a rare neuro-ophthalmologic disease (OMIM #615960) caused by mutations in the laminin subunit alpha-1 (*LAMA1*) gene and has an autosomal recessive pattern of inheritance [1, 2]. *LAMA1* encodes laminin subunit alpha 1, a cell adhesion molecule localized in basement membranes [3]. *Lama1*-conditional knockout mice show behavioral disorders and impaired cerebellum formation [4, 5]. PTBHS usually manifests in childhood as nonprogressive ataxia, intellectual disability, ocular motor apraxia, myopia, and retinal dystrophy. Cerebellar dysplasia, cerebellar cysts, cerebellar vermis hypoplasia, and splayed superior cerebellar peduncles have also been observed on brain imaging of patients with PTBHS [6].

Here, we report a rare case of a 65-year-old woman with mild ataxia and intellectual disability who was finally diagnosed with a novel homozygous variant in *LAMA1* resulting in PTBHS.

## Case Presentation

A 65-year-old Japanese woman with cerebellar malformation identified during a medical checkup was referred to our hospital for further evaluation. Her medical history was unremarkable. She was born to nonconsanguineous parents from neighboring towns and had healthy siblings. She had mild intellectual disability but had graduated junior high school and worked in factories. She was a slow runner during childhood and had difficulty walking in her early 60 s, with a slow, broadly based gait. At 64 years of age, she noticed memory loss and attention deficits. At 65 years of age, during a general medical examination, she was diagnosed with cognitive impairment and cerebellar hypoplasia on brain magnetic resonance imaging (MRI). She denied having any muscle weakness or dyspnea. She was subsequently admitted to our hospital for further evaluation.

**Fig. 1:**
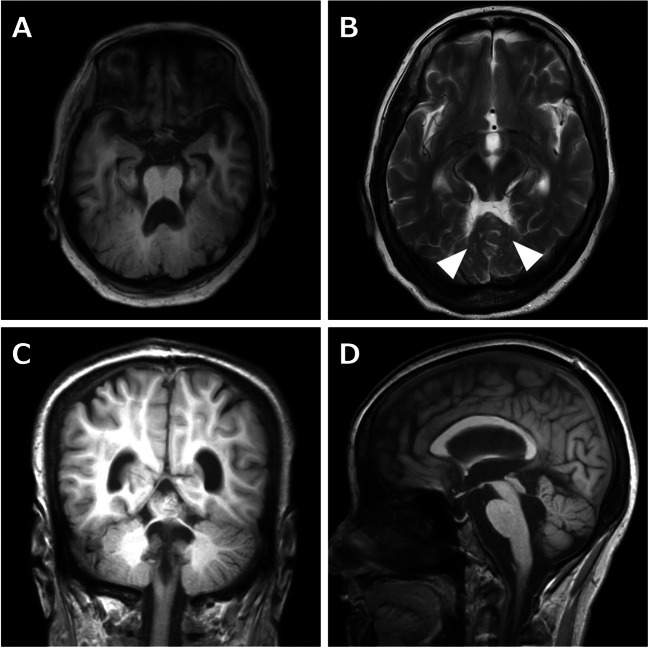
Magnetic resonance images (MRI): **A** Axial T1-weighted MRI, **B** Axial T2-weighted MRI, **C** Coronal T1-weighted MRI, and **D** Sagittal T1-weighted MRI. These images show elongated superior cerebellar peduncles without thickness or straight shape (**A**), multiple cerebellar cysts (**B**), splayed superior cerebellar peduncles (**C**), vermis hypoplasia, and rhomboid shaped fourth ventricle (**D**)

Neurological examination revealed vision loss in the left eye, left conductive hearing loss, impaired smooth pursuit of eye movement, mild cerebellar ataxia in the trunk and limbs, and hyperreflexia in the lower extremities. She also had a cataract in her left eye, but myopia and retinal involvement were unremarkable. The results of cognitive function assessment were as follows: 22/30 points on the Mini-Mental Scale Examination; 16/30 points on the Montreal Cognitive Assessment; and visual IQ: 75, performance IQ: 71, and full-scale IQ: 70 on the Wechsler Adult Intellectual Scale third edition. Blood test results revealed no abnormal findings. Brain MRI revealed multiple cysts in the antero-superior vermis and posterior-superior aspects of the hemispheres, cerebellar dysplasia, vermis hypoplasia, rhomboid shaped fourth ventricle, splayed superior cerebellar peduncles, and short pons (Fig. 1); however, supratentorial anomalies were absent. Diffusion tensor imaging showed preservation of the superior cerebellar peduncles decussate. Brain SPECT imaging, computed tomography, and whole-exome sequencing and filtering analysis revealed cerebellar hypoperfusion, absent cystic lesions or situs inversus, and a rare homozygous variant of c.1711_1712del in *LAMA1* (NM_005559.4), which lead to p. Ala571Profs*8 (NP_005550.2), respectively. Based on these clinical and genetic test results, the patient was finally diagnosed with PTBHS.

## Discussion

This report describes a rare case of PTBHS with a novel homozygous variant of *LAMA1* diagnosed in late adulthood. The patient presented with a mild and slowly progressive cognitive and motor symptoms and was diagnosed with PTBHS after a brain anomaly was identified on MRI scanning during her annual medical checkup.

Almost all patients with PTBHS experience a variable degree of developmental delay and ataxia, with the mean age of diagnosis being 3.1 years [6]. In this case, the patient had a limited educational and was not athletic. Cerebellar ataxia was minimal and did not affect standing or walking. *LAMA1* variants previously identified in patients with PTBHS include compound heterozygous or homozygous single nucleotide variant of either nonsense or frameshift variants, multiexon deletions, or duplications [6]. Although these mutations are thought to be associated with loss of function, the precise pathophysiology and phenotypic variability of *LAMA1* mutations remain unclear. The frameshift variant c.1711_1712del is absent from in-house databases and has been reported as a compound heterozygous in a patient without ataxia and ocular anomalies from a Chinese cohort including intellectual disability and developmental delay [7]. Although c.1711_1712del in *LAMA1* is considered a novel variant in PTBHS, further case studies are needed to determine its relationship with the milder and late-onset phenotype.

Although dystroglycanopathies and *GPR56*-related encephalopathy are also characterized by cerebellar cysts, PTBHS can be confirmed via neuroimaging (cortical malformation such as polymicrogyria) and symptom evaluation (muscle weakness and severe epilepsy) [8, 9]. Joubert syndrome (JS) is another disorder associated with cerebellar dysplasia. The age of JS onset is reported to vary, presenting any time from childhood to adulthood, with multiple prognoses. Brain MRI of a patient with JS revealed a peculiar constellation of mid-hindbrain malformations termed as the “molar tooth sign” that is sometimes misdiagnosed as a false positive in patients with different brain malformations [10]. In PTBHS, the brain MRI is characterized by multiple cortical–subcortical cysts in the antero-superior vermis and posterior-superior aspects of the hemispheres associated with dysplasia, vermis hypoplasia, and abnormal configuration of the fourth ventricle without brain stem or supratentorial abnormalities [9]. In this case, the superior cerebellar peduncles were elongated, thin, and oblique, making it easily distinguishable from the characteristic findings of JS [10].

## Data Availability

No datasets were generated or analysed during the current study.
